# Stresses and Displacements in Functionally Graded Materials of Semi-Infinite Extent Induced by Rectangular Loadings

**DOI:** 10.3390/ma5020210

**Published:** 2012-01-30

**Authors:** Hong-Tian Xiao, Zhong-Qi Yue

**Affiliations:** 1 Shandong Key Laboratory of Civil Engineering Disaster Prevention & Mitigation, Shandong University of Science and Technology, Shandong, Qingdao 266590, China; E-Mail: xiaohongtian@tsinghua.org.cn; 2 Department of Civil Engineering, The University of Hong Kong, Hong Kong, China

**Keywords:** functionally graded materials, FGM, elasticity, multilayered solids, Yue’s solution, Kelvin solution, stress analysis, numerical integration

## Abstract

This paper presents the stress and displacement fields in a functionally graded material (FGM) caused by a load. The FGM is a graded material of Si_3_N_4_-based ceramics and is assumed to be of semi-infinite extent. The load is a distributed loading over a rectangular area that is parallel to the external surface of the FGM and either on its external surface or within its interior space. The point-load analytical solutions or so-called Yue’s solutions are used for the numerical integration over the distributed loaded area. The loaded area is discretized into 200 small equal-sized rectangular elements. The numerical integration is carried out with the regular Gaussian quadrature. Weak and strong singular integrations encountered when the field points are located on the loaded plane, are resolved with the classical methods in boundary element analysis. The numerical integration results have high accuracy.

## 1. Introduction

Functionally graded materials (FGMs), also known as graded materials, are generally multi-phase composites with continuously varying mechanical properties. They are primarily used as coatings and interfacial zones to reduce stresses resulting from the material property mismatch, to improve the surface properties and to provide protection against severe loading, thermal and chemical environments. At present, FGMs are usually associated with particulate composites where the volume fraction of particles varies in one or several directions. With the wide use of FGMs in engineering, much attention has been paid to the mechanical behavior of FGMs due to different loadings.

Significant efforts have been made in the study of the mechanical responses of FGMs. These studies include the investigation of elastic fields, crack and contact problems in FGMs. For example, Gibson [1], Booker *et al.* [2], Oner [3] and Butter *et al.* [4] analyzed the elastic behavior of non-homogeneous half-spaces. Kassir [5], Giannakopoulos *et al.* [6] and Suresh [7] discussed the contact problems between punches and graded materials. Chen *et al.* [8] presented a semianalytical approach to solve the time-dependent response of a multilayered pavement. Delale *et al.* [9] analyzed the fracture mechanics of crack problems in FGMs. Chan *et al.* [10] and Martin *et al.* [11] presented Green’s functions for 2D and 3D exponentially-graded elastic solids, respectively. Birman *et al.* [12] presented a review of the principal developments in FGMs and the critical areas where further research is needed for a successful implementation of FGM in design. In particular, Carrera and his co-workers [13,14,15] developed the unified formulation for analysis of classical layered structures and extended it for FGM structures by using a set of functions which are indicated as thickness functions.

More recently, Xiao *et al.* [16] developed a numerical method in analyzing the contact between the rectangular rigid plate and the graded material of semi-infinite extent. Xiao *et al.* [17] further developed the numerical method for analysis of the elastic fields in heterogeneous rocks due to reservoir water impoundment. The numerical method has high accuracy and efficiency by using the generalized Kelvin solution of a multilayered medium of infinite extent, which is adoptable to a layered medium of infinite extent.

In this paper, the numerical method is used for the analysis of elastic behaviors of FGM half-space. Referring to the results in Pender *et al.* [18], the graded material of Si_3_N_4_-based ceramics of semi-infinite extent is used as the FGM for the stress and displacement analysis. The rectangular loading area is parallel to the boundary of the semi-infinite FGM space and the uniform normal loads are chosen. The displacements and stresses induced in the graded materials are presented. The comparison of elastic fields is made for two different positions of the rectangular loads.

## 2. Numerical Method for Analysis of Mechanical States in FGMs

### 2.1. The Point-Load Solution Suitable for the FGM

Yue [19] presented the point-load analytical solutions for multilayer elastic solids. This point-load solution is an extension of the classical Kelvin solution for a point-load in a homogeneous elastic space and is for the stress and displacement fields in a layered elastic solid of infinite extent caused by the action of point loads. Each layer is a homogeneous elastic solid of finite thickness and infinite lateral extension. The total number of the dissimilar elastic layers is an arbitrary integer. The internal layers adhere to the first homogeneous elastic solid of upper semi-infinite extent and the last homogeneous elastic solid of lower semi-infinite extent. The interface between any two connected dissimilar layers is planar and fully bonded. All the layer interfaces are parallel to each other.

**Figure 1:**
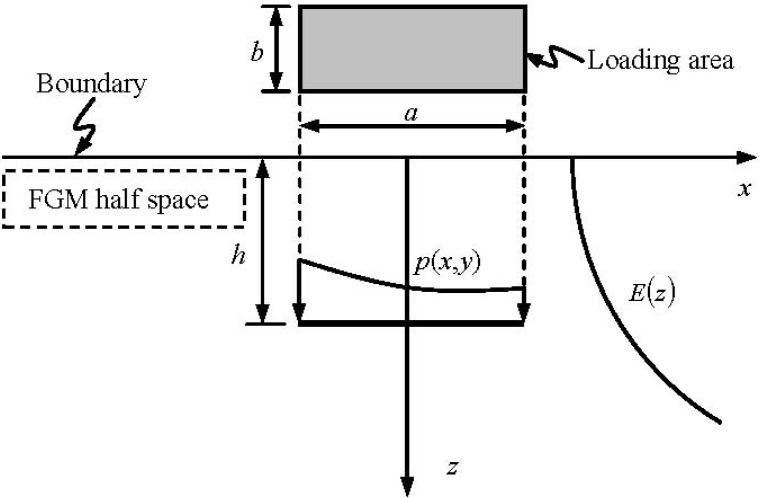
Functionally Graded Material (FGM) half-space subjected to loads on a rectangular area (*a* = 2 mm, *b* = 1 mm).

The FGM shown in Figure 1 has its isotropic elastic properties variable in depth and the layered technique is used along the depth. For this FGM problem of semi-infinite extent, the shear modulus of the first solid of upper semi-infinite extent is assigned a zero value (or an infinitesimal value, e.g., 10^–15^ MPa). Consequently, the first elastic solid becomes a void space of upper semi-infinite extent. The Yue’s solution for a point load in a multilayered elastic solid of infinite extent is automatically degenerated into the generalized Mindlin solution for a point load in a layered half-space.

### 2.2. The Numerical Method for Analysis of the FGM due to Distributed Loadings

By using Yue’s solution, Xiao *et al.* [16,20] presented the numerical analysis of contact problems between rigid punch and the graded materials and elastic fields in heterogeneous rocks induced by reservoir water impoundment, respectively. The accuracy and effectiveness of the numerical integration method have been verified by comparing the numerical results with the existing analytical ones. The numerical integration method is again used in the analysis of the stress and displacement fields in the FGM as shown in Figure 1. It is also noted that Yue *et al.* [21] used the similar numerical integration method and analyzed the effects of tire-pavement contact pressure distributions on the response of asphalts concrete pavements.

**Figure 2:**
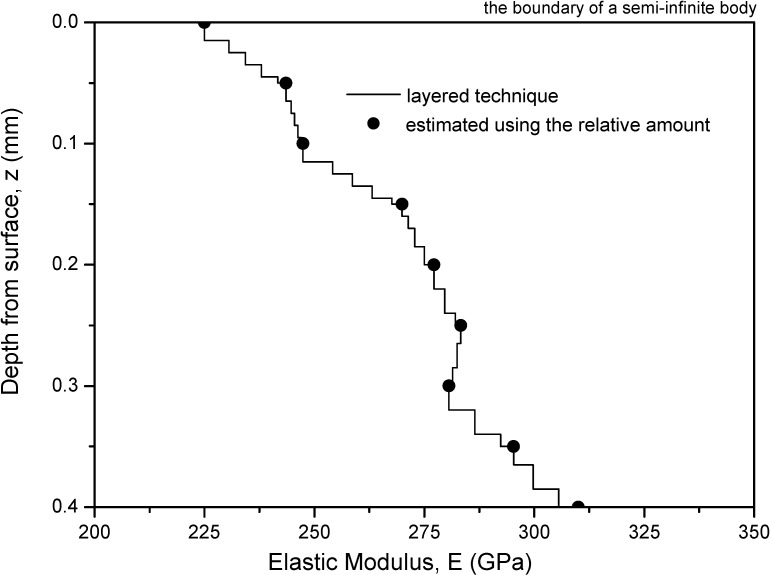
Variation of elastic modulus in layers for actual measured modulus.

Therefore, the mathematical formulation and computational procedures of the numerical integration method are not presented here in detail. Basically, the numerical integration method needs to discretize an FGM layer as a system of *n* number of fully bonded dissimilar sublayers. Thus, it uses Yue’s solution [19] for the elastostatic field in a layered solid of infinite extent due to the action of concentrated point loads. Figure 2 shows the discretization approach for FGMs. The Yue’s solution is used as the point-load solution to replace the classical Mindlin point-load solution in conventional numerical integration method for a homogeneous medium of semi-infinite extent. As a result, any FGMs with arbitrary property gradient in depth can be examined using this numerical method.

As shown in Figure 1, the surface *S* of the layered medium of semi-infinite extent is subjected to loads in the *x*, *y* and/or *z* directions. The total number of the dissimilar layers is an arbitrary integer. The stresses and displacements at any points of the layered medium are described as

(1) $$\sigma_{ij}(Q)=\int_{S}\sigma_{ijk}^{*}(Q,P)t_{k}(P)dS(P),\;i,j,k=x,y,z,$$

(2) $$u_{i}(Q)=\int_{S}u_{ik}^{*}(Q,P)t_{k}(P)dS(P),\;i,k=x,y,z$$

where σ_ijk_*(Q, P) and u_ik_*(Q, P) are the point-load solutions of the layered medium; σ_ijk_*(Q, P) are stresses for the field point *Q* due to the unit force along the *k* direction at the source point *P*; u_ik_*(Q, P) displacements for the field point *Q* along the *i* direction due to the unit force along the *k* direction at the source point *P*; t_k_(P) is the traction at the source point *P*.

Due to the irregular shapes of a loading area and the non-uniform distribution of the traction t_k_(P), the integrals shown in Expressions (1) and (2) cannot be integrated into analytical forms. The 2D integrals in (1) and (2) have to be calculated numerically. A discretization technique, similar to that used in boundary element methods [16], is adopted. The loading area *S* is discretized into quadrilateral elements. In each element, the interpolation functions between the global and local coordinates are introduced. Thus, the integral on each element is executed in local coordinates and is calculated by using the regular Gaussian quadrature.

If the source point *Q* is located at the integral element, the integrand of Expression (1) is strongly singular and the integrand of Expression (2) is weakly singular. The strongly singular integral of Expression (1) is calculated by using the indirect method and the weakly singular integral of Expression (2) is calculated by dividing an element into several triangular sub-elements and using the coordinate transformation.

## 3. Displacements and Stresses in FGMs under Rectangular Loadings

### 3.1. General

In most of existing solutions to problems relating to FGMs, it is assumed that the material is isotropic, the Poisson’s ratio is constant, and Young’s (or shear) modulus is either an exponential or a power function of a space variable. In the studies described in Delale *et al.* [9], the shear modulus is assumed to be μ = μ_0_exp(αy) where y = 0 is either the boundary of the half plane or the plane of the crack. In Kassir [5], it is assumed that μ = μ_0_׀y׀^m^, (0 < m < 1).Generally, it is easy to obtain the analytical solutions of FGMs for the above-mentioned assumption of the material properties. Actually, the properties of FGMs are distributed in complex forms. Thus, the proposed numerical method is used much effectively to analyze the mechanical response of the actual FGMs because it can also take into account the depth variations in both Young’s modulus and Poisson’s ratio.

Herein, the Si_3_N_4_-based materials given in Pender *et al.* [18] are further used for the stress analysis. The Si_3_N_4_-based graded materials were fabricated with controlled, unidirectional gradients in elastic modulus from the surface to the interior. The elastic parameters shown in Figure 2 were estimated by using several photomicrographs and image analysis software. The FGM had a constant Poisson’s ratio 0.22 and its elastic modulus is described by a piecewise linear interpolation as follows

(3) $$\begin{array}{l} E(z)=225.01+370.6z,\;0\leq z\leq 0.05\text{mm}\\ E(z)=243.54+76.4(z-0.05)\;,\;0.05\leq z\leq 0.10\text{mm}\\ E(z)=247.36+450.8(z-0.1),\;0.10\leq z\leq 0.15\text{mm}\\ E(z)=269.90+145.0(z-0.15),\;0.15\leq z\leq 0.20\text{mm}\\ E(z)=277.20+122.2(z-0.20),\;0.20\leq z\leq 0.25\text{mm}\\ E(z)=283.26-53.4(z-0.25),\;0.25\leq z\leq 0.30\text{mm}\\ E(z)=280.59+294.1(z-0.30)\text{, }0.30\leq z\leq 0.40\text{mm}\\ E(z)=310.0,\text{ z}\geq 0.40\text{mm} \end{array}$$

where the unit of elastic modulus is GPa and *z* is the depth coordinate, as shown in Figure 2.

The FGM from the depth z = 0 mm to 0.4 mm is discretized into 30 thin layers, as shown in Figure 2. Each layer has a constant modulus from Equation (3). The FGM from the depth z = 0.4 mm to ∞ is modeled as a homogeneous elastic solid of lower half-space extent. The above-mentioned FGMs will be referred to as Case 1. For reference and comparison, a homogeneous medium of semi-infinite extent is chosen and will be referred to as Case 2. The Young’s modulus and the Poisson’s ratio of the homogeneous elastic solid are assumed to be 225.1 GPa and 0.22, respectively, (*i.e.*, *E*_0_ = 225.1 GPa and ν = 0.22). This modulus value is equal to the average value of the FGM modulus.

**Figure 3:**
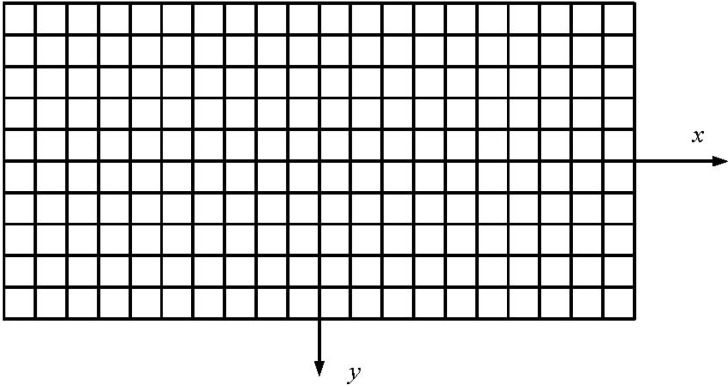
The mesh of loading area with 200 eight-noded elements and 661 nodes.

The loaded area (*a* × *b*) is a rectangular, where a and b are the length and width, respectively. It is assumed that *a* = 2 mm and *b* = 1 mm. The loaded area is parallel to the boundary of FGMs at *z* = 0 mm. The loaded area is subjected to the uniform vertical load *p*(*x*,*y*), where –a/2 ≤ x ≤ a/2 and –b/2 ≤ y ≤ b/2. The rectangular loaded area is discretized into the mesh of 200 eight-noded elements and 661 nodes, as shown in Figure 3.

Lankford [22] measured hardness and compressive strength of several strong ceramics from room temperature to 1,000 °C and gave the tensile and compressive strengths of the ceramic Si_3_N_4_. It can be found that the tensile strength of Si_3_N_4_ is 810 MPa and the compressive strength of Si_3_N_4_ is more than 1.5 GPa. Herein, it is assumed that *p*(*x*,*y*) = 100 MPa. For this case, the FGMs may be in the elastic state and the proposed method can be used for analysis of the displacement and stress fields.

For the FGM half-space subjected to the rectangular loading, the distribution characteristics of the elastic fields at the loaded plane are in general similar to those of the elastic fields in two bonded rocks of infinite extent induced by the rectangular loadings [17]. In Xiao *et al.* [17], the closed-form solutions were presented for the elastic fields in two bonded rocks of infinite extent induced by the uniform rectangular loadings. The continuities of the stresses and displacements across the loading plane and the material interface were discussed in detail. In the ensuing, distributions of the elastic fields for Cases 1 and 2 at different depths are presented and discussed in detail.

### 3.2. The Loading Area at h = 0 mm

**Figure 4:**
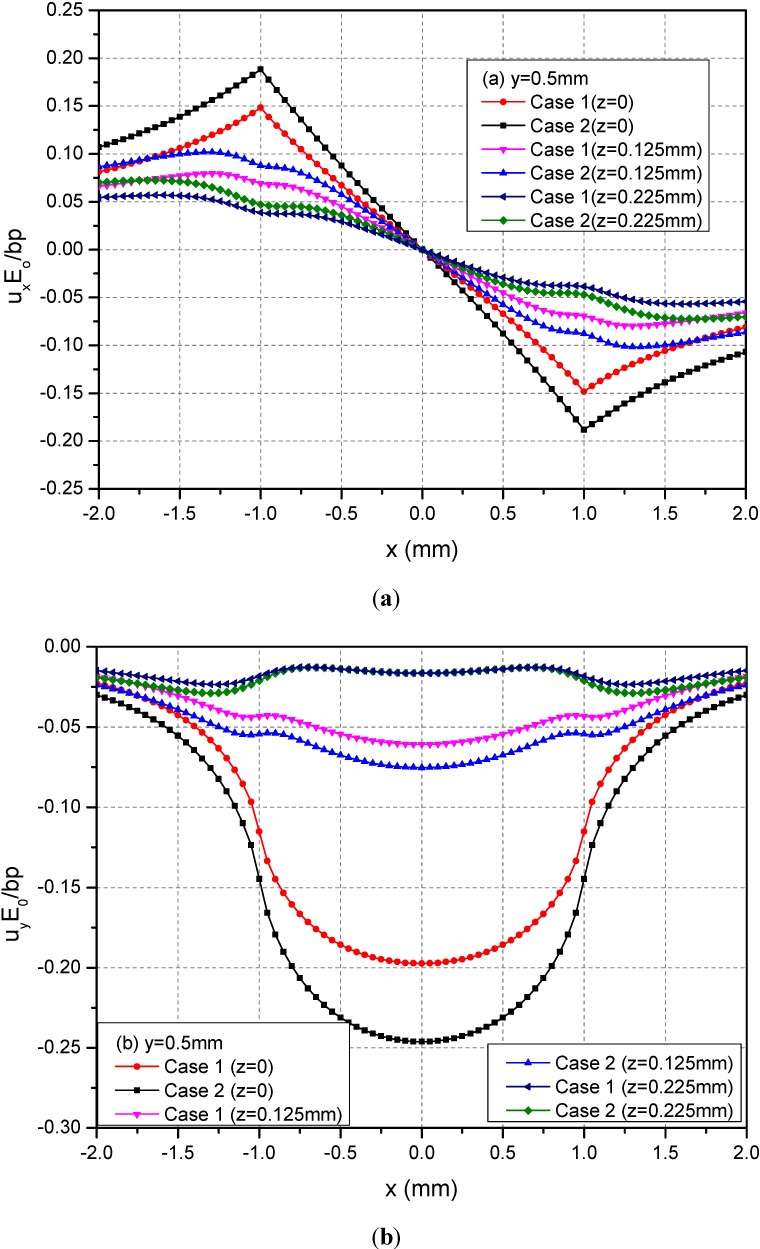

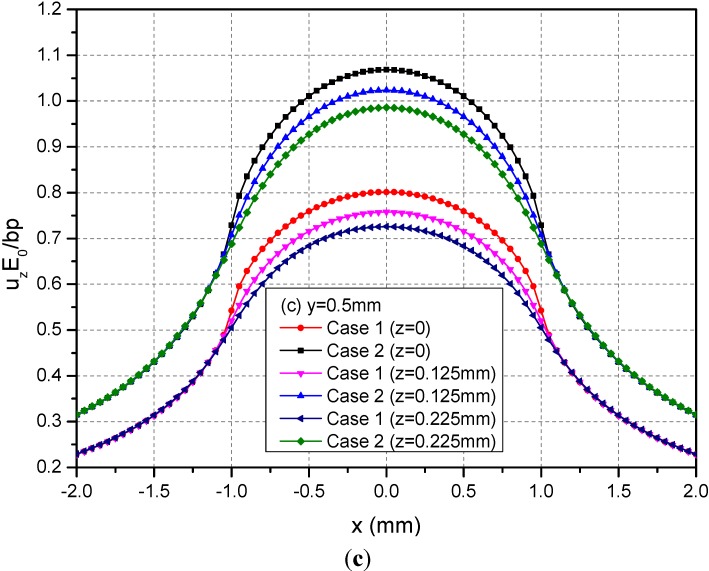
Normalized displacements (**a**) u_x_; (**b**) u_y_; (**c**) u_z_ along *y* = 0.5 mm at z = 0, 0.125, 0.225 mm for the loading area h = 0 mm.

The rectangular loading on the plane h = 0 mm is examined in this section. In this case, the loading is applied on the boundary of the FGMs of semi-infinite extent. Figure 4 shows the variations of the three normalized displacements u_x_, u_y_ and u_z_ along y = 0.5 mm at the depths z = 0, 0.125 and 0.225 mm. From these figures, it can be observed that the absolute values of displacements decrease as the depth increased for the two cases. At a given depth, the absolute values of the displacements of Case 1 are smaller than the ones of Case 2.

**Figure 5:**
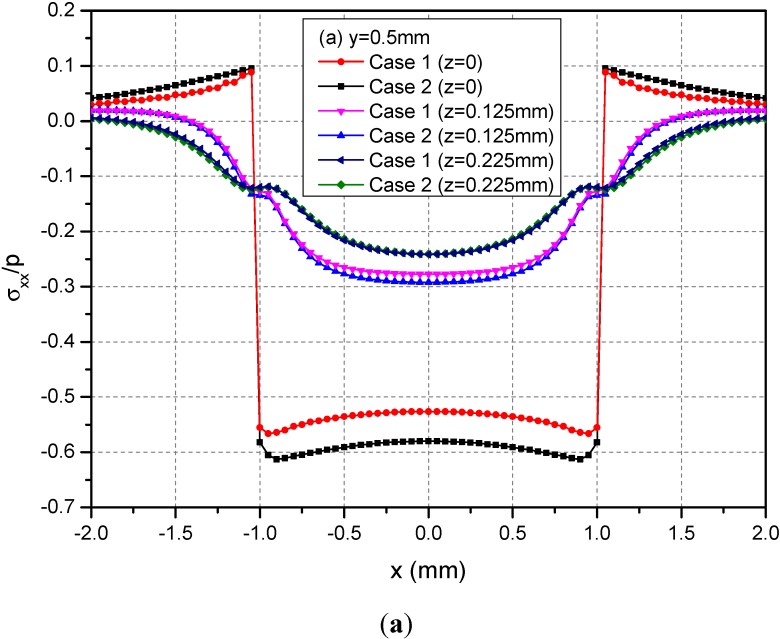

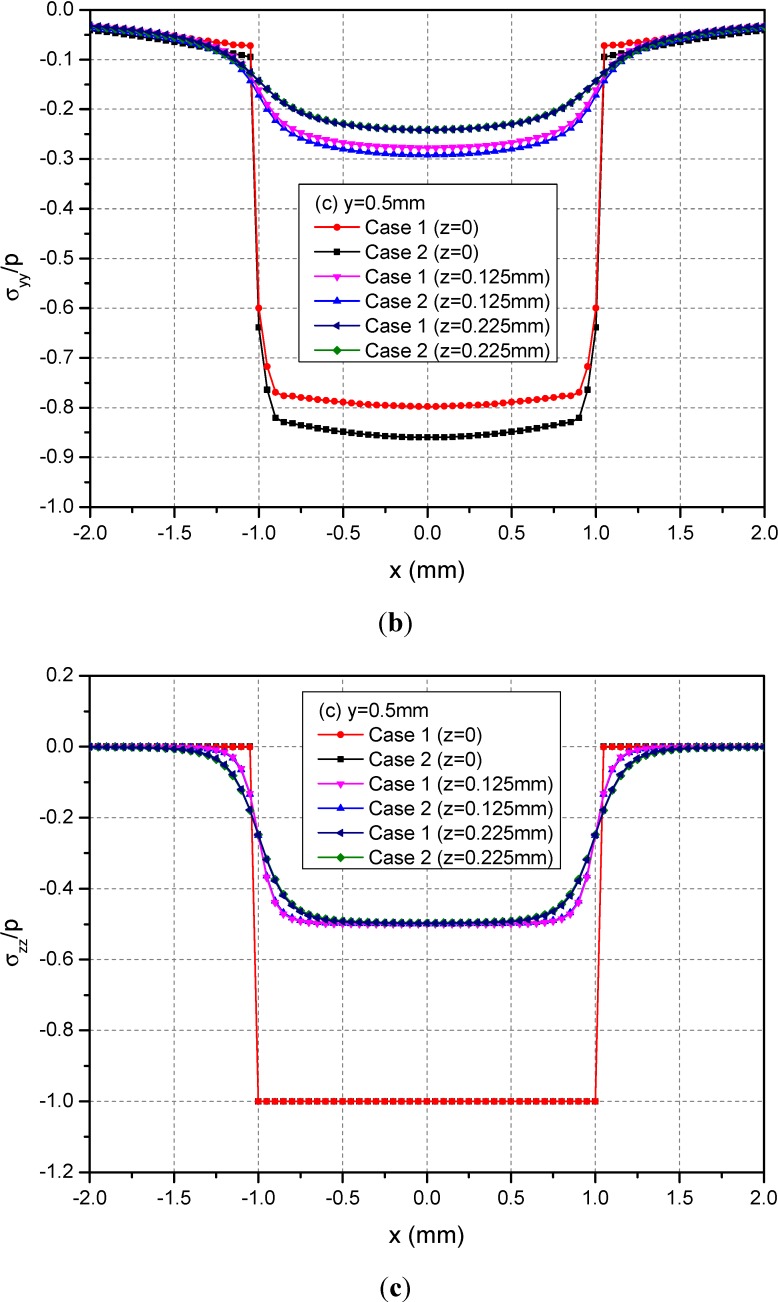
Normalized stresses (**a**) σ_xx_; (**b**) σ_yy_; (**c**) σ_zz_ along y = 0.5 mm at z = 0, 0.125, 0.225 mm for the loading area h = 0 mm.

**Figure 6:**
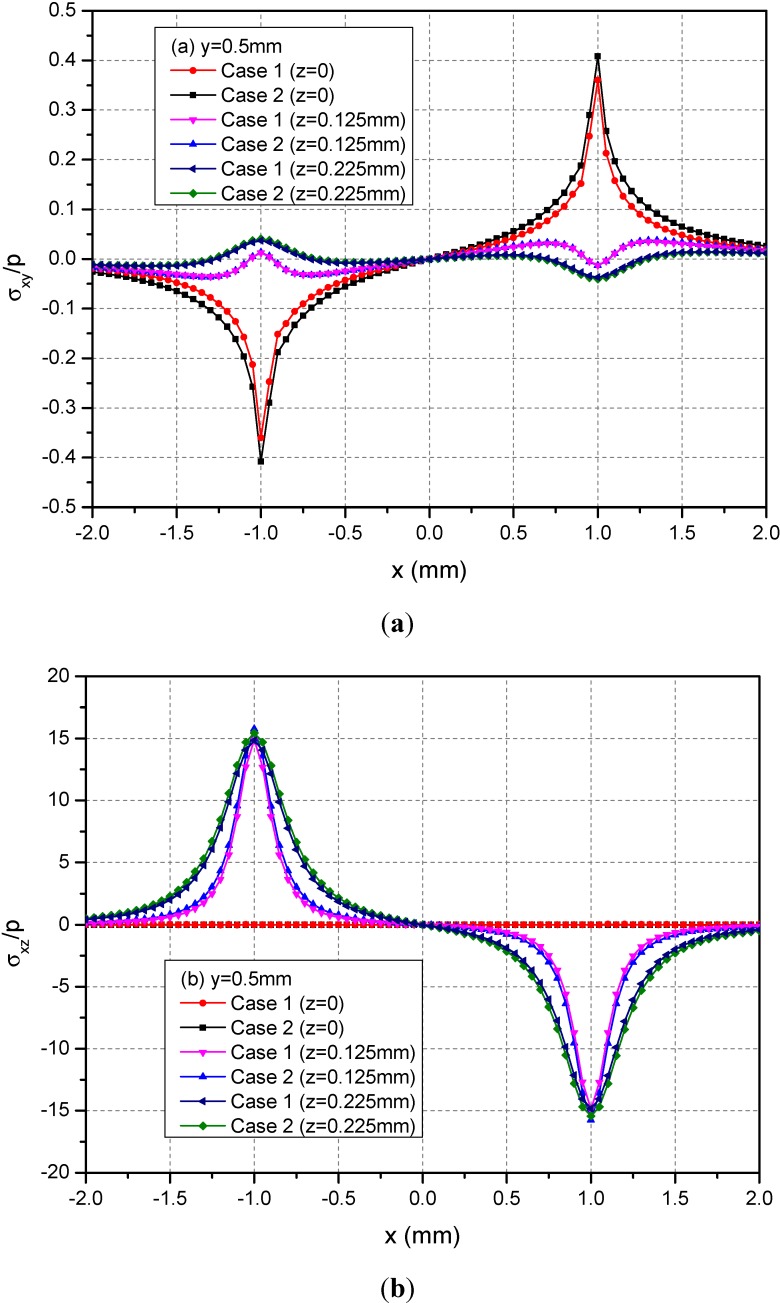

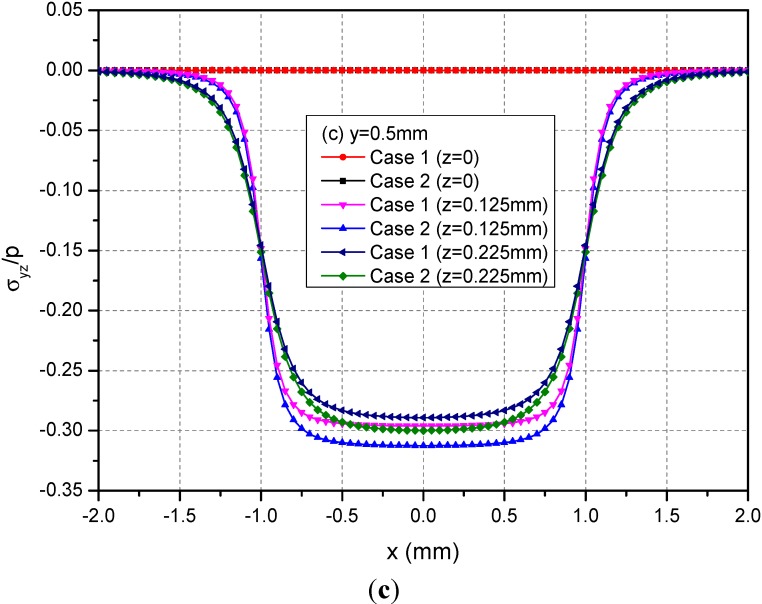
Normalized stresses (**a**) σ_xx_; (**b**) σ_yy_; (**c**) σ_zz_ along y = 0.5 mm at z = 0, 0.125, 0.225 mm for the loading area h = 0 mm.

Figure 5 shows the variations of the three normalized normal stresses σ_xx_, σ_yy_ and σ_zz_ along y = 0.5 mm at depths z = 0, 0.125 and 0.225 mm. It can be observed that the normal stresses are discontinuous at the points x = ± 1.0 mm and z = 0 mm across the loading area. At a given depth and among −1.0 mm < x < 1.0 mm, the absolute values of σ_xx_ and σ_yy_ for Case 1 are smaller than the those of the corresponding normal stresses for Case 2. At the depth z = 0.225 mm, this influence is not obvious. Figure 6 shows the variations of the three normalized shear stresses σ_xy_, σ_xz_ and σ_yz_ along y = 0.5 mm at the depths z = 0, 0.125 or 0.225 mm. It can be observed that there are no obvious influences of material heterogeneities on the distributions of stresses except σ_yz_. At the depth z = 0.225 mm and among −1.0 mm < x < 1.0 mm, the σ_yz_ values have obvious differences between Cases 1 and 2.

### 3.3. The Loading Area at h = 0.13 mm

**Figure 7:**
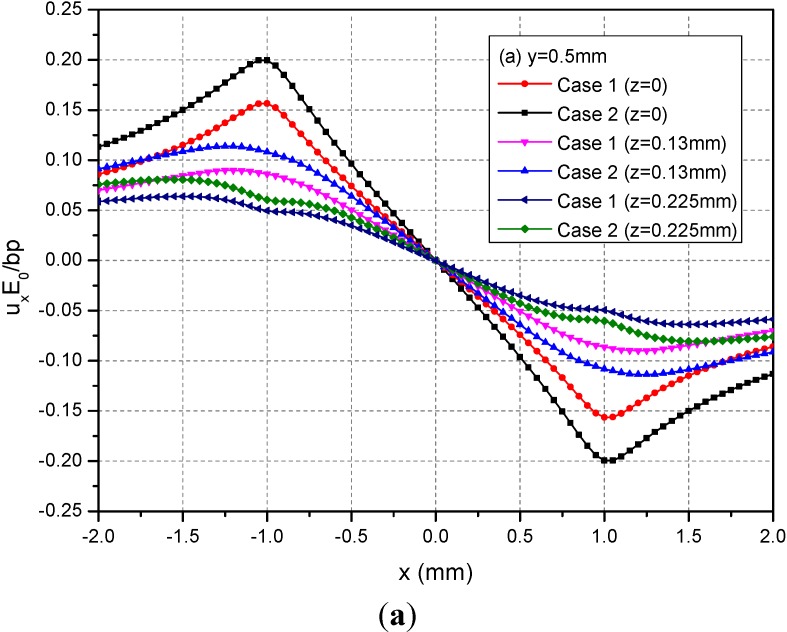

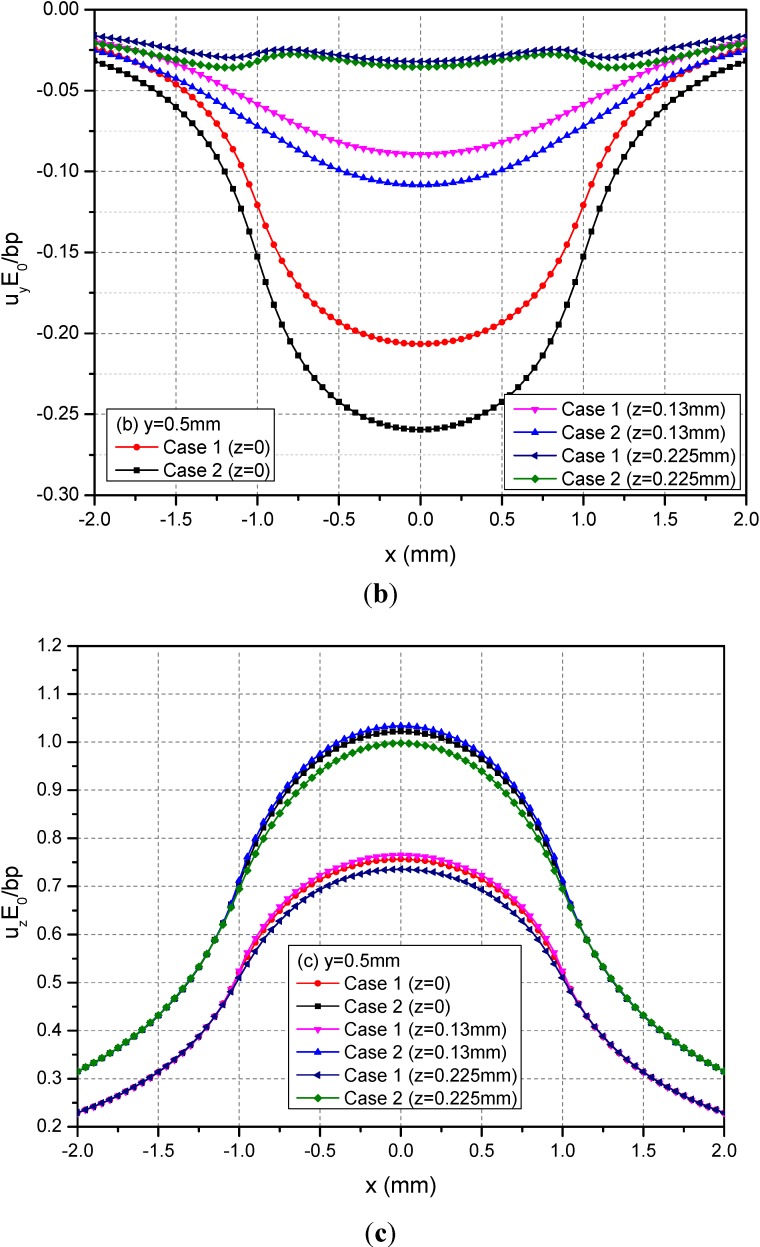
Normalized displacements (**a**) u_x_; (**b**) u_y_; (**c**) u_z_ along y = 0.5 mm at z = 0, 0.13, 0.225 mm for the loading area h = 0.13 mm.

The rectangular loading on the plane h = 0.13 mm is examined in this section. In this case, the loading is applied within the FGMs of semi-infinite extent. Figure 7 shows the variations of normalized displacements along y = 0.5 mm for the depths z = 0, 0.13 and 0.225 mm. It can be observed that the absolute values of the three displacements at a given depth for Case 1 are smaller than those of the corresponding displacements for Case 2. The phenomenon is more obvious in the neighborhood of x = ±1.0 mm for u_x_ and among −1.0 mm ≤ x ≤ 1.0 mm for u_y_ and u_z_.

**Figure 8:**
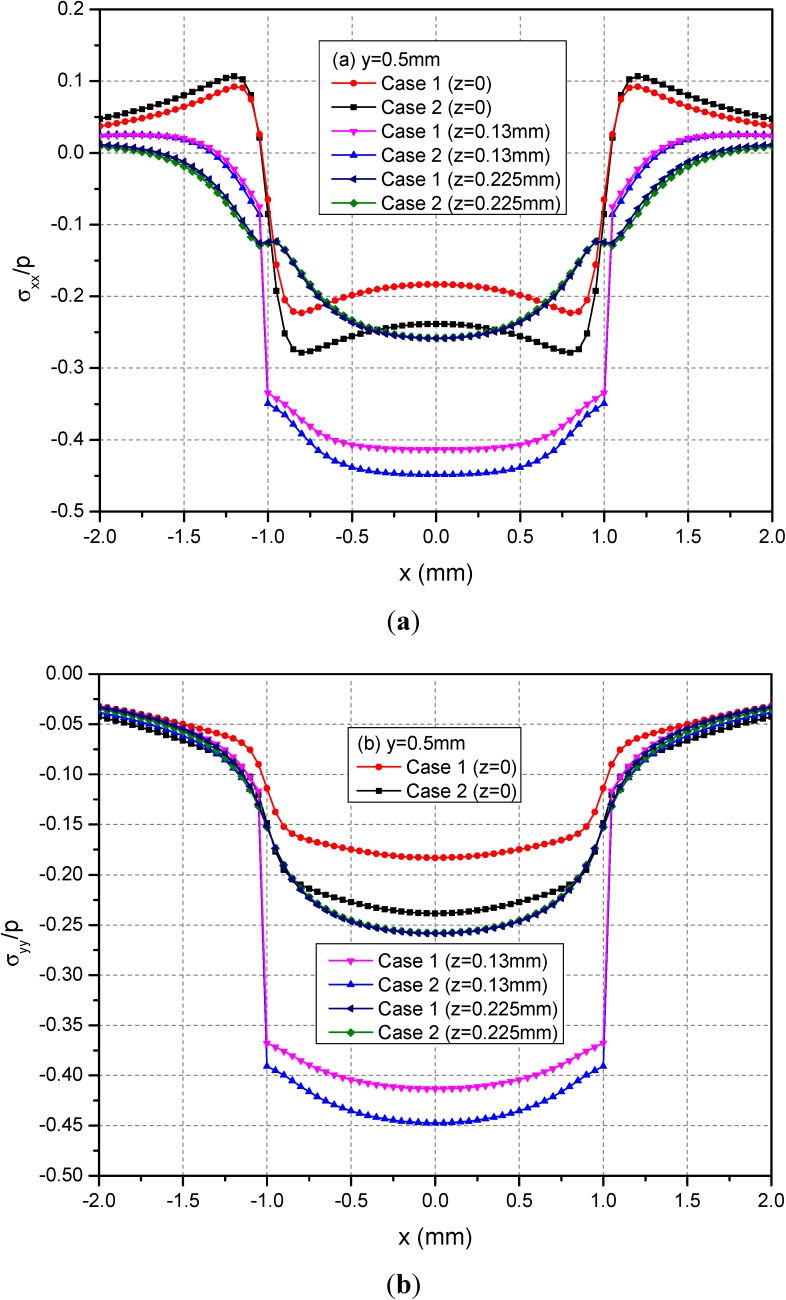

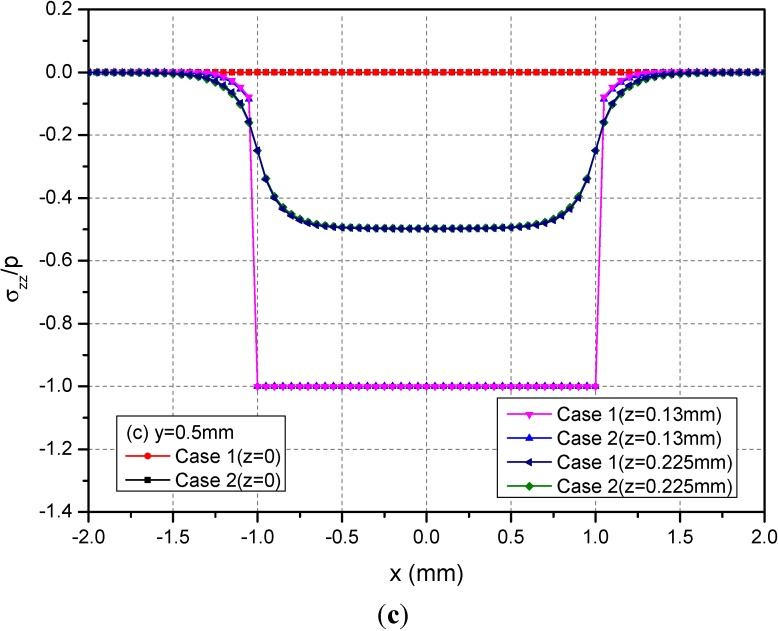
Normalized stresses (**a**) σ_xx_; (**b**) σ_yy_; (**c**) σ_zz_ along y = 0.5 mm at z = 0, 0.13, 0.225 mm for the loading area h = 0.13 mm.

**Figure 9:**
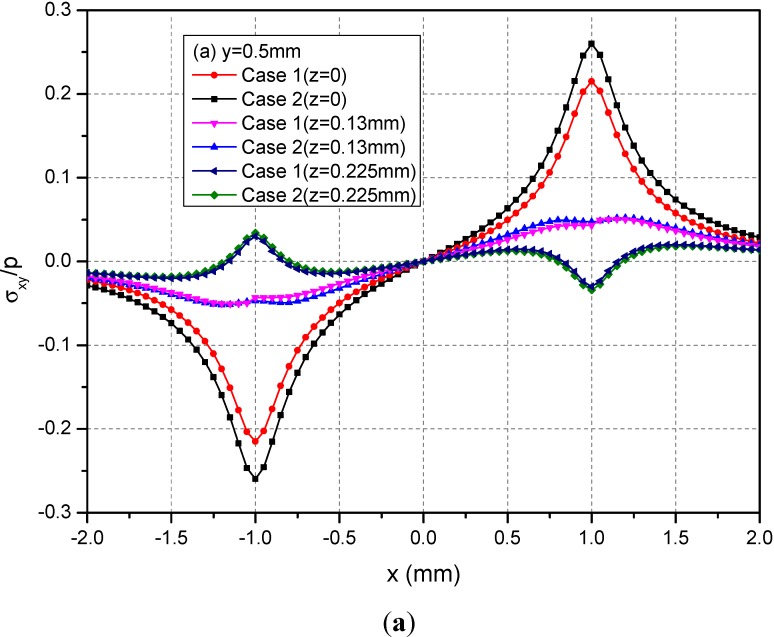

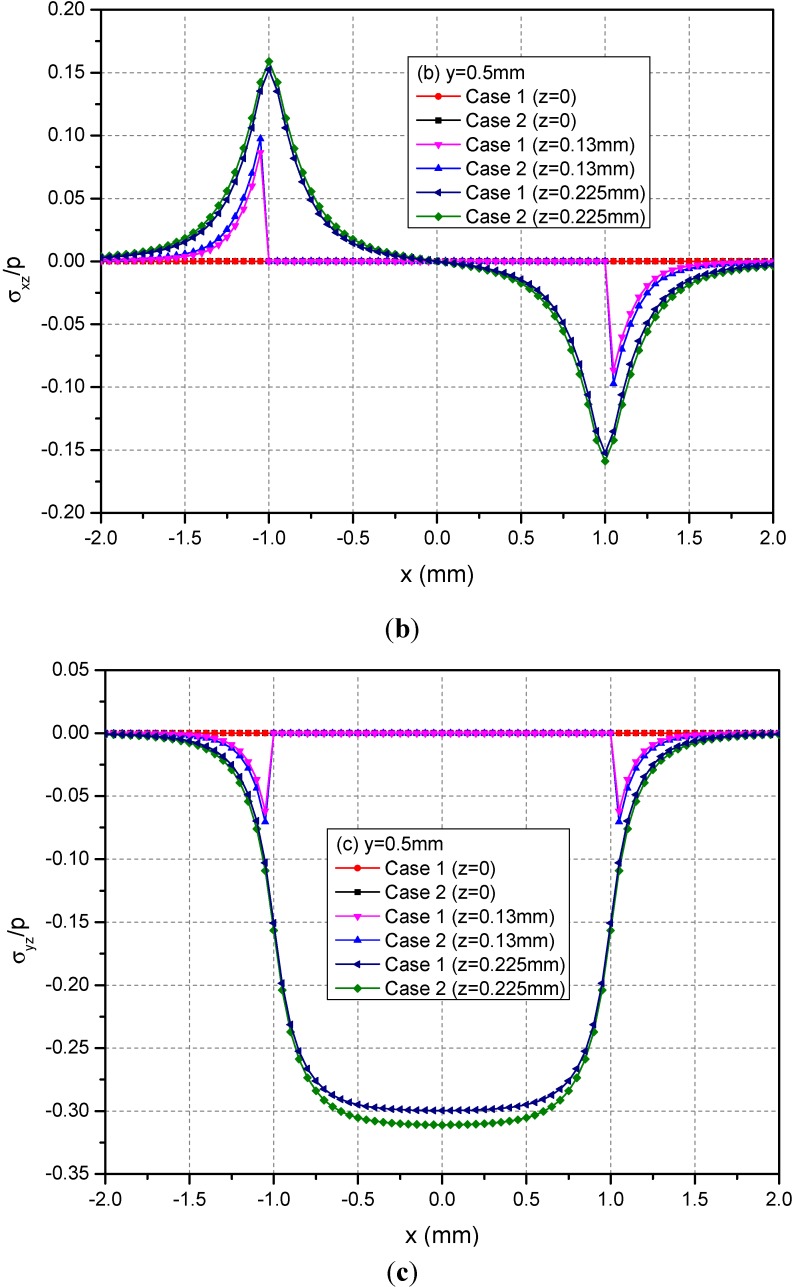
Normalized stresses (**a**) σ_xy_; (**b**) σ_xz_; (**c**) σ_yz_ along y = 0.5 mm at z = 0, 0.13, 0.225 mm for the loading area h = 0.13 mm.

Figure 8 shows the variations of the three normalized normal stresses along y = 0.5 mm at the depths z = 0, 0.13 and 0.225 mm. It can be found that the normal stresses σ_xx_ and σ_yy_ for Cases 1 and 2 have obvious differences for −1.0 mm ≤ x ≤ 1.0 mm at the depths z = 0 mm and 0.13 mm while the stresses has negligible differences at z = 0.225 mm. However, the values of σ_zz_ at any depths for Cases 1 and 2 have no obvious differences. Figure 9 shows the variations of the three normalized shear stresses along y = 0.5 mm for different depths z = 0, 0.13 and 0.225 mm. It can be found that there are small differences between Cases 1 and 2 except the shear stress σ_xy_. The σ_xy_ values have large differences between Cases 1 and 2.

### 3.4. Comparison of Elastic Fields for the Two Loading Positions

Among the displacement components, the u_z_ values for h = 0.13 mm are smaller than the ones for h = 0 mm. This is because the materials above the loading plane h = 0.13 mm constrain the deformation of the FGMs. However, u_x_ and u_y_ have small differences for two loading positions.

For two loading positions, σ_xx_, σ_yy_ and σ_zz_ are the maximums at loading plane positions h = 0 mm and h = 0.13 mm, respectively. Away from the loading plane positions, the values of these stress components become small. For the two loading positions, the absolute values of σ_xy_ are the maximums at z = 0 mm, *i.e.*, the boundary of semi-infinite extent. At x = ±1.0 mm, σ_xz_ = 0 and σ_yz_ = 0 for the loading position h = 0 mm while σ_xz_ and σ_yz_ has a jump for the loading position h = 0.13 mm.

## 4. Conclusions

This paper has analyzed the stress and displacement fields in a functionally graded material of semi-infinite extent induced by rectangular loading. The FGM can have their elastic properties exhibiting the variation in depth while keeping constant in lateral directions. This paper has examined the theoretical stress and displacement fields in the Si_3_N_4_-based ceramics due to the rectangular loading. The displacements and stresses are presented and compared to those of a homogeneous elastic solid of semi-infinite extent. It was found that the heterogeneity of FGM has an evident influence on the elastic fields of the semi-infinite elastic solids. This capability to exactly calculate the complete elastic field induced in FGM is important to the understanding of the FGM mechanical behavior and in the design of FGM properties with depth.
